# [2-(2-Meth­oxy-1-naphtho­yl)phen­yl](1-naphth­yl)methanone

**DOI:** 10.1107/S1600536811042747

**Published:** 2011-10-29

**Authors:** G. Jagadeesan, K. Sethusankar, R. Sivasakthikumaran, Arasambattu K. Mohanakrishnan

**Affiliations:** a Department of Physics, Dr MGR Educational and Research Institute, Dr MGR University, Chennai 600 095, India; b Department of Physics, RKM Vivekananda College (Autonomous), Chennai 600 004, India; c Department of Organic Chemistry, University of Madras, Maraimalai Campus, Chennai 600 025, India

## Abstract

The title compound, C_29_H_20_O_3_, adopts an ‘S’ conformation with a dihedral angle of 68.5 (2)° beween the two acetone planes. The central phenyl ring forms dihedral angles of 83.8 (4) and 84.5 (4)° with the naphthalene and meth­oxy-substituted naphthalene mean planes, respectively. Both carbonyl-group O atoms deviate significantly from the naphthalene moiety and the meth­oxy-substituted naphthalene moiety [0.574 (1) and −1.053 (1) Å, respectively]. The crystal packing is stabilized by C—H⋯O inter­molecular inter­actions, generating *C*(7) chain and *R* ^2^_2_(10) graph-set motifs.

## Related literature

For the uses and biological importance of diketones, see: Bennett *et al.* (1999 ▶). For related structures, see: Tsumuki *et al.* (2011 ▶); Jagadeesan *et al.* (2011 ▶); Judas *et al.* (1995 ▶). For graph-set notation, see: Bernstein *et al.* (1995 ▶).
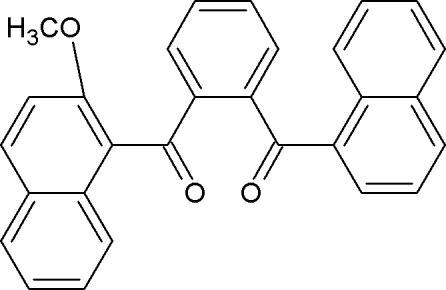


## Experimental

### 

#### Crystal data

C_29_H_20_O_3_
*M* *_r_* = 416.45
Monoclinic, 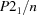

*a* = 8.3950 (3) Å
*b* = 8.9983 (4) Å
*c* = 28.5375 (11) Å
β = 97.188 (2)°
*V* = 2138.80 (15) Å^3^
*Z* = 4
Mo *K*α radiation
μ = 0.08 mm^−1^
*T* = 293 K
0.30 × 0.25 × 0.20 mm

#### Data collection

Bruker Kappa APEXII CCD diffractometer
26364 measured reflections
6299 independent reflections
3875 reflections with *I* > 2σ(*I*)
*R*_int_ = 0.036

#### Refinement

*R*[*F* ^2^ > 2σ(*F* ^2^)] = 0.049
*wR*(*F* ^2^) = 0.137
*S* = 1.00
6270 reflections
290 parameters
H-atom parameters constrained
Δρ_max_ = 0.23 e Å^−3^
Δρ_min_ = −0.17 e Å^−3^

### 

Data collection: *APEX2* (Bruker, 2008 ▶); cell refinement: *APEX2*; data reduction: *SAINT* (Bruker, 2008 ▶); program(s) used to solve structure: *SHELXS97* (Sheldrick, 2008 ▶); program(s) used to refine structure: *SHELXL97* (Sheldrick, 2008 ▶); molecular graphics: *ORTEP-3* (Farrugia, 1997 ▶); software used to prepare material for publication: *SHELXL97* and *PLATON* (Spek, 2009 ▶).

## Supplementary Material

Crystal structure: contains datablock(s) global, I. DOI: 10.1107/S1600536811042747/bt5676sup1.cif

Structure factors: contains datablock(s) I. DOI: 10.1107/S1600536811042747/bt5676Isup2.hkl

Supplementary material file. DOI: 10.1107/S1600536811042747/bt5676Isup3.cml

Additional supplementary materials:  crystallographic information; 3D view; checkCIF report

## Figures and Tables

**Table 1:** Hydrogen-bond geometry (Å, °)

*D*—H⋯*A*	*D*—H	H⋯*A*	*D*⋯*A*	*D*—H⋯*A*
C3—H3⋯O1^i^	0.93	2.49	3.392 (2)	164
C13—H13⋯O1^ii^	0.93	2.52	3.440 (2)	170
C27—H27⋯O2^iii^	0.93	2.51	3.262 (3)	138

Symmetry codes: (i) 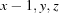; (ii) 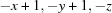; (iii) 
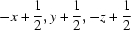
.

**Fig. 1.**
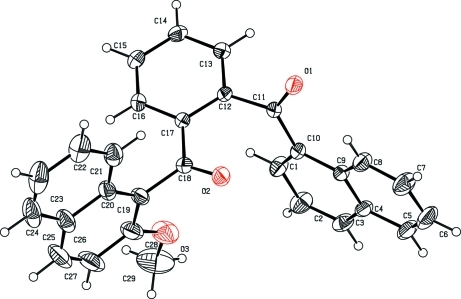
The molecular structure of the title compound with the atomic numbering scheme and displacement ellipsoids at the 30% probability level.

**Fig. 2.**
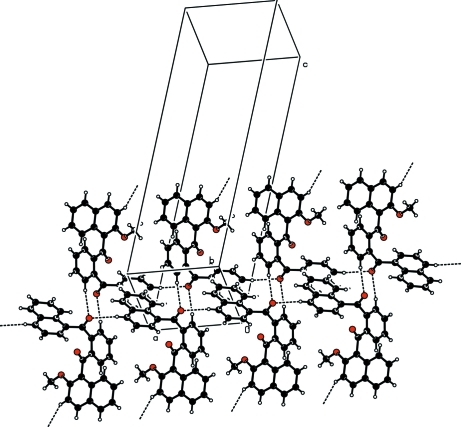
The packing arrangement of the title compound. Dashed lines indicates the C–H···O interactions. Symmetry code: (i) x - 1, y, z; (ii) -x + 1, -y + 1, -z; (iii) -x + 1/2, y + 1/2, -z + 1/2.

## supplementary crystallographic information

### Comment

Diketones are popular in organic synthesis, for their applications in biology and medicine. They are known to exhibit antioxidant, antitumour and antibacterial activities (Bennett *et al.*, 1999).

X-ray analysis confirms the molecular structure and atom connectivity of the title compound as illustrated in the Fig. 1. The central phenyl ring (C12–C17) of the compound forms dihedral angles of 83.8 (4)° and 84.5 (4)° with the naphthalene moiety (C1–C10) and methoxy substituted naphthalene moiety (C19–28), respectively. The central phenyl ring (C12–C17) forms dihedral angles of 69.7 (5)° and 11.1 (5)° with the mean planes of the ketone groups, (C10–C12/O1) and (C17–C19/O2), respectively. The dihedral angle between the methoxy substituted naphthalene moiety (C19–C28) and naphthlene moiety (C1–C10) is 64.2 (4)°.

The two benzene rings (C1—C4/C9/C10) and (C4–C9) are almost coplanar with a dihedral angle of 2.26 (6)° between them. The atoms C29, O2 and O3 are having deviations of 0.363 (3) Å, -1.053 (1)Å and 0.101 (1)Å from the mean plane of the methoxy substituted naphthalene ring (C19–C28), respectively. The atom O1 deviates by 0.574 (1)Å from the plane of the naphthlene ring (C1–C10). The C10–C11 and C18–C19 bond lengths of 1.49 (2)Å and 1.50 (2)Å respectively and can be considered as single C(*sp*^2^)–C(*sp*^2^) bond distances. The molecule possesses distorted S–conformation in which C19/C18/C17/C12/C11/C10/O1/O2 are in a single plane, which is determined by the dihedral angle of 68.5 (2)° beween the planes defined by C19/C18/C17/O2 and that through C10/C11/C12/O1 (Judas *et al.*, 1995).The title compound exhibits the structural similarities with the reported related structures (Tsumuki *et al.*, 2011 & Jagadeesan *et al.*, 2011).

The crystal packing is stabilized by C–H···O interactions (Table 1). The C3–H3···O1^i^ interaction generates a *C*(7) chain along the *a* axis and the C13–H13···O1^ii^ hydrogen bond generates *R*^2^_2_(10) graphset motifs (Bernstein *et al.*, 1995); the carbonyl-group O1 atom is involved in bifurcated hydrogen bonding. The Symmetry codes are: (i) *x* - 1, *y*, *z*; (ii) -*x* + 1, -*y* + 1, -*z*; (iii) -*x* + 1/2, *y* + 1/2, -*z* + 1/2. The packing view of the compound is shown in (Fig. 2).

### Experimental

To a stirred suspension of 1-(2-methoxy-1-naphthoyl)phenyl-1-naphthyl- 2-benzofuran (1 g, 3.22 mmol) in dry THF (20 ml), lead tetraaccetate (1.52 g, 3.42 mmol) was added and refluxed at 343 K for half an hour. The reaction mixture was then poured into water (200 ml) and extracted with ethyl acetate (2*x*20 ml), washed with brine solution and dried (Na_2_SO_4_). The removal of solvent *in vacuo* afforded crude product. The crude product upon crystallization from methanol furnished the tittle compound as a colorless solid.

### Refinement

Hydrogen atoms were placed in calculated positions with C–H = 0.93Å and 0.96Å and refined using a the riding model with fixed isotropic displacement parameters: *U*_iso_(H) = 1.5 *U*_eq_(C) for the methyl group and *U*_iso_(H) = 1.2 *U*_eq_(C) for other groups.

### Crystal data

**Table:**

C_29_H_20_O_3_	*F*(000) = 872
*M**_r_* = 416.45	*D*_x_ = 1.293 Mg m^−^^3^
Monoclinic, *P*2_1_/*n*	Mo *K*α radiation, λ = 0.71073 Å
Hall symbol: -P 2yn	Cell parameters from 3875 reflections
*a* = 8.3950 (3) Å	θ = 1.4–30.1°
*b* = 8.9983 (4) Å	µ = 0.08 mm^−^^1^
*c* = 28.5375 (11) Å	*T* = 293 K
β = 97.188 (2)°	Block, colourless
*V* = 2138.80 (15) Å^3^	0.30 × 0.25 × 0.20 mm
*Z* = 4	

### Data collection

**Table:**

Bruker Kappa APEXII CCD diffractometer	3875 reflections with *I* > 2σ(*I*)
Radiation source: fine-focus sealed tube	*R*_int_ = 0.036
graphite	θ_max_ = 30.1°, θ_min_ = 1.4°
ω scans	*h* = −10→11
26364 measured reflections	*k* = −12→12
6299 independent reflections	*l* = −40→40

### Refinement

**Table:**

Refinement on *F*^2^	Primary atom site location: structure-invariant direct methods
Least-squares matrix: full	Secondary atom site location: difference Fourier map
*R*[*F*^2^ > 2σ(*F*^2^)] = 0.049	Hydrogen site location: inferred from neighbouring sites
*wR*(*F*^2^) = 0.137	H-atom parameters constrained
*S* = 1.00	*w* = 1/[σ^2^(*F*_o_^2^) + (0.0541*P*)^2^ + 0.4431*P*] where *P* = (*F*_o_^2^ + 2*F*_c_^2^)/3
6270 reflections	(Δ/σ)_max_ < 0.001
290 parameters	Δρ_max_ = 0.23 e Å^−^^3^
0 restraints	Δρ_min_ = −0.17 e Å^−^^3^

### Special details

**Table:**

Geometry. All e.s.d.'s (except the e.s.d. in the dihedral angle between two l.s. planes) are estimated using the full covariance matrix. The cell e.s.d.'s are taken into account individually in the estimation of e.s.d.'s in distances, angles and torsion angles; correlations between e.s.d.'s in cell parameters are only used when they are defined by crystal symmetry. An approximate (isotropic) treatment of cell e.s.d.'s is used for estimating e.s.d.'s involving l.s. planes.
Refinement. Refinement of *F*^2^ against ALL reflections. The weighted *R*-factor *wR* and goodness of fit *S* are based on *F*^2^, conventional *R*-factors *R* are based on *F*, with *F* set to zero for negative *F*^2^. The threshold expression of *F*^2^ > σ(*F*^2^) is used only for calculating *R*-factors(gt) *etc*. and is not relevant to the choice of reflections for refinement. *R*-factors based on *F*^2^ are statistically about twice as large as those based on *F*, and *R*- factors based on ALL data will be even larger.

### Fractional atomic coordinates and isotropic or equivalent isotropic displacement parameters (Å^2^)

**Table:**

	*x*	*y*	*z*	*U*_iso_*/*U*_eq_	
C1	0.15231 (17)	0.63183 (17)	0.06709 (6)	0.0491 (4)	
H1	0.2003	0.7251	0.0696	0.059*	
C2	−0.01600 (18)	0.6217 (2)	0.06134 (7)	0.0585 (4)	
H2	−0.0780	0.7075	0.0606	0.070*	
C3	−0.08746 (17)	0.4876 (2)	0.05692 (6)	0.0523 (4)	
H3	−0.1989	0.4816	0.0521	0.063*	
C4	0.00426 (16)	0.35663 (17)	0.05949 (5)	0.0437 (3)	
C5	−0.0706 (2)	0.2162 (2)	0.05524 (7)	0.0606 (5)	
H5	−0.1820	0.2108	0.0499	0.073*	
C6	0.0158 (2)	0.0898 (2)	0.05872 (8)	0.0763 (6)	
H6	−0.0359	−0.0016	0.0553	0.092*	
C7	0.1836 (2)	0.0961 (2)	0.06743 (8)	0.0708 (5)	
H7	0.2426	0.0084	0.0707	0.085*	
C8	0.26131 (18)	0.22922 (17)	0.07110 (6)	0.0507 (4)	
H8	0.3728	0.2313	0.0768	0.061*	
C9	0.17494 (15)	0.36407 (15)	0.06640 (5)	0.0367 (3)	
C10	0.24751 (14)	0.50808 (15)	0.06903 (5)	0.0356 (3)	
C11	0.42381 (15)	0.52955 (15)	0.07111 (5)	0.0360 (3)	
C12	0.49178 (14)	0.68277 (14)	0.08141 (5)	0.0335 (3)	
C13	0.54639 (16)	0.75872 (17)	0.04437 (5)	0.0430 (3)	
H13	0.5386	0.7151	0.0146	0.052*	
C14	0.61245 (17)	0.89905 (18)	0.05125 (5)	0.0476 (4)	
H14	0.6483	0.9495	0.0261	0.057*	
C15	0.62522 (18)	0.96406 (17)	0.09500 (6)	0.0509 (4)	
H15	0.6696	1.0585	0.0995	0.061*	
C16	0.57230 (17)	0.88951 (16)	0.13223 (5)	0.0458 (3)	
H16	0.5815	0.9340	0.1619	0.055*	
C17	0.50522 (14)	0.74850 (14)	0.12605 (5)	0.0351 (3)	
C18	0.45546 (15)	0.66581 (15)	0.16664 (5)	0.0383 (3)	
C19	0.44871 (19)	0.74572 (17)	0.21243 (5)	0.0473 (4)	
C20	0.5827 (2)	0.74335 (17)	0.24785 (5)	0.0514 (4)	
C21	0.7276 (2)	0.67257 (19)	0.24152 (6)	0.0592 (4)	
H21	0.7352	0.6202	0.2138	0.071*	
C22	0.8574 (3)	0.6795 (2)	0.27548 (7)	0.0767 (6)	
H22	0.9527	0.6329	0.2706	0.092*	
C23	0.8476 (4)	0.7560 (3)	0.31743 (8)	0.0913 (8)	
H23	0.9369	0.7610	0.3402	0.110*	
C24	0.7105 (4)	0.8225 (2)	0.32528 (7)	0.0904 (8)	
H24	0.7059	0.8718	0.3537	0.108*	
C25	0.5721 (3)	0.8193 (2)	0.29106 (6)	0.0696 (5)	
C26	0.4270 (4)	0.8903 (3)	0.29698 (8)	0.0916 (8)	
H26	0.4179	0.9379	0.3255	0.110*	
C27	0.3004 (3)	0.8919 (3)	0.26284 (9)	0.0871 (7)	
H27	0.2061	0.9403	0.2679	0.104*	
C28	0.3114 (2)	0.8202 (2)	0.21948 (7)	0.0639 (5)	
C29	0.0565 (3)	0.9120 (4)	0.18389 (11)	0.1213 (11)	
H29A	0.0035	0.8871	0.2108	0.182*	
H29B	−0.0166	0.8989	0.1555	0.182*	
H29C	0.0913	1.0137	0.1863	0.182*	
O1	0.51456 (11)	0.43299 (12)	0.06066 (4)	0.0542 (3)	
O2	0.42381 (13)	0.53442 (11)	0.16317 (4)	0.0503 (3)	
O3	0.19135 (16)	0.81810 (18)	0.18259 (6)	0.0838 (4)	

### Atomic displacement parameters (Å^2^)

**Table:**

	*U*^11^	*U*^22^	*U*^33^	*U*^12^	*U*^13^	*U*^23^
C1	0.0369 (7)	0.0401 (8)	0.0693 (11)	−0.0038 (6)	0.0026 (7)	−0.0014 (7)
C2	0.0351 (7)	0.0559 (10)	0.0836 (13)	0.0070 (7)	0.0041 (7)	0.0041 (9)
C3	0.0290 (6)	0.0676 (11)	0.0598 (10)	−0.0062 (7)	0.0033 (6)	0.0063 (8)
C4	0.0375 (7)	0.0522 (9)	0.0420 (8)	−0.0141 (6)	0.0069 (6)	0.0021 (7)
C5	0.0463 (8)	0.0634 (11)	0.0731 (12)	−0.0245 (8)	0.0110 (8)	0.0027 (9)
C6	0.0722 (12)	0.0518 (11)	0.1073 (17)	−0.0308 (10)	0.0205 (11)	−0.0035 (11)
C7	0.0673 (11)	0.0412 (9)	0.1065 (16)	−0.0086 (8)	0.0215 (10)	0.0029 (10)
C8	0.0452 (8)	0.0419 (8)	0.0661 (11)	−0.0069 (6)	0.0115 (7)	0.0009 (7)
C9	0.0355 (6)	0.0399 (7)	0.0349 (7)	−0.0074 (5)	0.0058 (5)	−0.0014 (6)
C10	0.0304 (6)	0.0382 (7)	0.0378 (7)	−0.0053 (5)	0.0025 (5)	−0.0038 (6)
C11	0.0329 (6)	0.0389 (7)	0.0368 (7)	−0.0056 (5)	0.0064 (5)	−0.0080 (6)
C12	0.0274 (5)	0.0355 (7)	0.0384 (7)	−0.0051 (5)	0.0066 (5)	−0.0052 (6)
C13	0.0413 (7)	0.0510 (9)	0.0375 (7)	−0.0073 (6)	0.0087 (6)	−0.0048 (6)
C14	0.0480 (8)	0.0503 (9)	0.0466 (9)	−0.0121 (7)	0.0137 (6)	0.0055 (7)
C15	0.0554 (9)	0.0406 (8)	0.0590 (10)	−0.0179 (7)	0.0154 (7)	−0.0045 (7)
C16	0.0531 (8)	0.0402 (8)	0.0456 (8)	−0.0131 (6)	0.0121 (6)	−0.0104 (6)
C17	0.0345 (6)	0.0339 (7)	0.0379 (7)	−0.0054 (5)	0.0089 (5)	−0.0053 (5)
C18	0.0382 (7)	0.0380 (7)	0.0399 (7)	−0.0026 (5)	0.0098 (6)	−0.0026 (6)
C19	0.0647 (9)	0.0411 (8)	0.0397 (8)	−0.0047 (7)	0.0207 (7)	−0.0036 (6)
C20	0.0812 (11)	0.0394 (8)	0.0356 (8)	−0.0111 (8)	0.0146 (7)	−0.0007 (6)
C21	0.0761 (11)	0.0516 (10)	0.0475 (10)	−0.0066 (9)	−0.0009 (8)	0.0005 (8)
C22	0.0938 (14)	0.0626 (12)	0.0669 (13)	−0.0111 (11)	−0.0165 (11)	0.0125 (10)
C23	0.142 (2)	0.0629 (13)	0.0579 (13)	−0.0311 (14)	−0.0316 (14)	0.0148 (11)
C24	0.173 (3)	0.0596 (13)	0.0351 (10)	−0.0294 (15)	−0.0001 (13)	−0.0008 (9)
C25	0.1247 (17)	0.0506 (10)	0.0361 (9)	−0.0165 (11)	0.0207 (10)	−0.0034 (7)
C26	0.156 (2)	0.0736 (14)	0.0544 (12)	−0.0021 (15)	0.0489 (15)	−0.0182 (11)
C27	0.1146 (18)	0.0784 (15)	0.0797 (16)	0.0114 (13)	0.0576 (14)	−0.0141 (12)
C28	0.0750 (11)	0.0614 (11)	0.0618 (11)	0.0034 (9)	0.0339 (10)	−0.0052 (9)
C29	0.0917 (17)	0.142 (3)	0.139 (3)	0.0545 (17)	0.0487 (16)	0.023 (2)
O1	0.0376 (5)	0.0475 (6)	0.0803 (8)	−0.0058 (4)	0.0181 (5)	−0.0230 (6)
O2	0.0664 (7)	0.0387 (6)	0.0471 (6)	−0.0084 (5)	0.0125 (5)	0.0002 (5)
O3	0.0645 (8)	0.0969 (11)	0.0935 (11)	0.0205 (8)	0.0227 (8)	−0.0104 (9)

### Geometric parameters (Å, °)

**Table:**

C1—C10	1.3678 (19)	C15—H15	0.9300
C1—C2	1.405 (2)	C16—C17	1.3904 (18)
C1—H1	0.9300	C16—H16	0.9300
C2—C3	1.347 (2)	C17—C18	1.4804 (19)
C2—H2	0.9300	C18—O2	1.2131 (16)
C3—C4	1.405 (2)	C18—C19	1.499 (2)
C3—H3	0.9300	C19—C28	1.370 (2)
C4—C5	1.410 (2)	C19—C20	1.415 (2)
C4—C9	1.4233 (18)	C20—C21	1.404 (2)
C5—C6	1.345 (3)	C20—C25	1.422 (2)
C5—H5	0.9300	C21—C22	1.366 (3)
C6—C7	1.401 (3)	C21—H21	0.9300
C6—H6	0.9300	C22—C23	1.392 (3)
C7—C8	1.362 (2)	C22—H22	0.9300
C7—H7	0.9300	C23—C24	1.340 (4)
C8—C9	1.411 (2)	C23—H23	0.9300
C8—H8	0.9300	C24—C25	1.421 (3)
C9—C10	1.4298 (18)	C24—H24	0.9300
C10—C11	1.4864 (17)	C25—C26	1.404 (3)
C11—O1	1.2170 (16)	C26—C27	1.349 (4)
C11—C12	1.5069 (18)	C26—H26	0.9300
C12—C13	1.3842 (19)	C27—C28	1.409 (3)
C12—C17	1.3961 (18)	C27—H27	0.9300
C13—C14	1.383 (2)	C28—O3	1.364 (2)
C13—H13	0.9300	C29—O3	1.417 (3)
C14—C15	1.371 (2)	C29—H29A	0.9600
C14—H14	0.9300	C29—H29B	0.9600
C15—C16	1.376 (2)	C29—H29C	0.9600
			
C10—C1—C2	121.74 (14)	C15—C16—H16	119.6
C10—C1—H1	119.1	C17—C16—H16	119.6
C2—C1—H1	119.1	C16—C17—C12	119.18 (12)
C3—C2—C1	119.91 (15)	C16—C17—C18	120.59 (12)
C3—C2—H2	120.0	C12—C17—C18	120.18 (11)
C1—C2—H2	120.0	O2—C18—C17	120.42 (12)
C2—C3—C4	120.81 (13)	O2—C18—C19	120.46 (13)
C2—C3—H3	119.6	C17—C18—C19	119.10 (12)
C4—C3—H3	119.6	C28—C19—C20	120.71 (15)
C3—C4—C5	120.80 (13)	C28—C19—C18	119.15 (15)
C3—C4—C9	120.24 (13)	C20—C19—C18	120.14 (13)
C5—C4—C9	118.96 (14)	C21—C20—C19	122.59 (14)
C6—C5—C4	121.39 (15)	C21—C20—C25	118.58 (17)
C6—C5—H5	119.3	C19—C20—C25	118.81 (17)
C4—C5—H5	119.3	C22—C21—C20	120.98 (18)
C5—C6—C7	119.99 (16)	C22—C21—H21	119.5
C5—C6—H6	120.0	C20—C21—H21	119.5
C7—C6—H6	120.0	C21—C22—C23	120.3 (2)
C8—C7—C6	120.71 (17)	C21—C22—H22	119.8
C8—C7—H7	119.6	C23—C22—H22	119.8
C6—C7—H7	119.6	C24—C23—C22	120.6 (2)
C7—C8—C9	120.89 (15)	C24—C23—H23	119.7
C7—C8—H8	119.6	C22—C23—H23	119.7
C9—C8—H8	119.6	C23—C24—C25	121.4 (2)
C8—C9—C4	117.97 (12)	C23—C24—H24	119.3
C8—C9—C10	124.32 (12)	C25—C24—H24	119.3
C4—C9—C10	117.70 (12)	C26—C25—C20	118.2 (2)
C1—C10—C9	119.52 (11)	C26—C25—C24	123.7 (2)
C1—C10—C11	118.01 (12)	C20—C25—C24	118.1 (2)
C9—C10—C11	122.38 (12)	C27—C26—C25	122.34 (18)
O1—C11—C10	123.24 (12)	C27—C26—H26	118.8
O1—C11—C12	117.88 (11)	C25—C26—H26	118.8
C10—C11—C12	118.53 (11)	C26—C27—C28	119.8 (2)
C13—C12—C17	119.41 (12)	C26—C27—H27	120.1
C13—C12—C11	117.14 (12)	C28—C27—H27	120.1
C17—C12—C11	123.43 (11)	O3—C28—C19	115.73 (15)
C14—C13—C12	120.49 (13)	O3—C28—C27	124.13 (18)
C14—C13—H13	119.8	C19—C28—C27	120.1 (2)
C12—C13—H13	119.8	O3—C29—H29A	109.5
C15—C14—C13	120.21 (14)	O3—C29—H29B	109.5
C15—C14—H14	119.9	H29A—C29—H29B	109.5
C13—C14—H14	119.9	O3—C29—H29C	109.5
C14—C15—C16	119.94 (14)	H29A—C29—H29C	109.5
C14—C15—H15	120.0	H29B—C29—H29C	109.5
C16—C15—H15	120.0	C28—O3—C29	119.16 (19)
C15—C16—C17	120.77 (14)		
			
C10—C1—C2—C3	−0.9 (3)	C11—C12—C17—C16	178.86 (12)
C1—C2—C3—C4	2.1 (3)	C13—C12—C17—C18	−176.75 (12)
C2—C3—C4—C5	179.49 (17)	C11—C12—C17—C18	1.67 (19)
C2—C3—C4—C9	−0.6 (2)	C16—C17—C18—O2	−166.95 (14)
C3—C4—C5—C6	−178.45 (18)	C12—C17—C18—O2	10.20 (19)
C9—C4—C5—C6	1.6 (3)	C16—C17—C18—C19	11.53 (19)
C4—C5—C6—C7	0.9 (3)	C12—C17—C18—C19	−171.32 (12)
C5—C6—C7—C8	−1.9 (3)	O2—C18—C19—C28	−94.73 (19)
C6—C7—C8—C9	0.2 (3)	C17—C18—C19—C28	86.79 (18)
C7—C8—C9—C4	2.3 (2)	O2—C18—C19—C20	85.03 (18)
C7—C8—C9—C10	−178.96 (16)	C17—C18—C19—C20	−93.45 (17)
C3—C4—C9—C8	176.89 (14)	C28—C19—C20—C21	−177.85 (16)
C5—C4—C9—C8	−3.2 (2)	C18—C19—C20—C21	2.4 (2)
C3—C4—C9—C10	−1.9 (2)	C28—C19—C20—C25	0.2 (2)
C5—C4—C9—C10	177.98 (14)	C18—C19—C20—C25	−179.52 (14)
C2—C1—C10—C9	−1.7 (2)	C19—C20—C21—C22	176.34 (16)
C2—C1—C10—C11	174.95 (15)	C25—C20—C21—C22	−1.7 (2)
C8—C9—C10—C1	−175.73 (15)	C20—C21—C22—C23	0.7 (3)
C4—C9—C10—C1	3.0 (2)	C21—C22—C23—C24	0.7 (3)
C8—C9—C10—C11	7.8 (2)	C22—C23—C24—C25	−1.0 (3)
C4—C9—C10—C11	−173.44 (12)	C21—C20—C25—C26	179.73 (17)
C1—C10—C11—O1	−160.14 (15)	C19—C20—C25—C26	1.6 (2)
C9—C10—C11—O1	16.4 (2)	C21—C20—C25—C24	1.4 (2)
C1—C10—C11—C12	12.87 (19)	C19—C20—C25—C24	−176.73 (16)
C9—C10—C11—C12	−170.62 (12)	C23—C24—C25—C26	−178.3 (2)
O1—C11—C12—C13	65.58 (17)	C23—C24—C25—C20	−0.1 (3)
C10—C11—C12—C13	−107.81 (14)	C20—C25—C26—C27	−1.8 (3)
O1—C11—C12—C17	−112.87 (15)	C24—C25—C26—C27	176.4 (2)
C10—C11—C12—C17	73.74 (17)	C25—C26—C27—C28	0.2 (4)
C17—C12—C13—C14	−0.6 (2)	C20—C19—C28—O3	178.49 (15)
C11—C12—C13—C14	−179.09 (12)	C18—C19—C28—O3	−1.7 (2)
C12—C13—C14—C15	0.3 (2)	C20—C19—C28—C27	−1.9 (3)
C13—C14—C15—C16	0.1 (2)	C18—C19—C28—C27	177.88 (16)
C14—C15—C16—C17	−0.2 (2)	C26—C27—C28—O3	−178.7 (2)
C15—C16—C17—C12	−0.1 (2)	C26—C27—C28—C19	1.7 (3)
C15—C16—C17—C18	177.11 (14)	C19—C28—O3—C29	−169.66 (19)
C13—C12—C17—C16	0.44 (19)	C27—C28—O3—C29	10.7 (3)

### Hydrogen-bond geometry (Å, °)

**Table:**

*D*—H···*A*	*D*—H	H···*A*	*D*···*A*	*D*—H···*A*
C3—H3···O1^i^	0.93	2.49	3.392 (2)	164
C13—H13···O1^ii^	0.93	2.52	3.440 (2)	170
C27—H27···O2^iii^	0.93	2.51	3.262 (3)	138

Symmetry codes: (i) *x*−1, *y*, *z*; (ii) −*x*+1, −*y*+1, −*z*; (iii) −*x*+1/2, *y*+1/2, −*z*+1/2.
